# Jump in Elective Total Hip and Knee Arthroplasty Numbers at Age 65 Years: Evidence for Moral Hazard?

**DOI:** 10.5435/JAAOSGlobal-D-22-00035

**Published:** 2022-03-22

**Authors:** Kelsey A. Rankin, Isaac G. Freedman, Harold G. Moore, Scott J. Halperin, Lee E. Rubin, Jonathan N. Grauer

**Affiliations:** From the Yale Department of Orthopedics and Rehabilitation, New Haven, CT (Ms. Rankin, Mr. Freedman, Mr. Halperin, Dr. Rubin, and Dr. Grauer), and Weill Cornell Medical College, New York, NY (Mr. Moore).

## Abstract

Total knee arthroplasty (TKA) and total hip arthroplasty (THA) are outstanding options for many older patients at the discretion of the patient and the surgeon. As patients turn 65 years, greater than 98% of the US population becomes eligible for Medicare, and this represents a time of changing healthcare coverage for many.

**Methods:**

Patients undergoing elective TKA and THA were abstracted from the 2012 to 2018 National Surgical Quality Improvement Program database. Incidences of TKA and THA (combined and separate) were plotted and assessed by age. To assess factors associated with who “delayed” surgery until 65 years, demographic and preoperative characteristics, and postoperative adverse events were compared for the 2 years above and the 2 years below the 65-year-old mark with multivariate analysis. Significance was set at *P* < 0.05.

**Results:**

In total, 515,139 TKA and THA patients were identified (62.04% TKA and 37.95% THA). When the number of procedures was plotted by age, a discontinuity in the bell-shaped curve was noted at age 65 years. Highlighting this finding, the changes in percent population between 63 and 64 years was −1.52%, between 64 and 65 years was +15.36%, and between 65 and 66 years was −2.32%. Relative to those who were 63 and 64 years (n = 36,511), those who were 65 and 66 years (n = 41,671) were more likely to be female, be non-Hispanic White, have a lower body mass index, and have a lower functional status but were not different in the preoperative American Society of Anesthesiologists class.

**Conclusion:**

In this large national sample, there was a clear step increase in undergoing TKA or THA once patients reached the age of 65 years (Medicare eligibility). This discontinuity in the bell-shaped curve may be evidence for a moral hazard in healthcare markets. Although factors in decision-making were not assessed, there were demographic factors associated with this step finding.

The US current model for health care is a decentralized combination of public and private options that predominantly are accessed by—and depend on—employment.^1^ However, this model shifts at age 65 years when >98% of the American population is able to access universal health care by Medicare eligibility.^2^

There are many variables that may be affected by this insurance change at 65 years, such as benefits, deductibles, and more.^3^ Overall, it has been shown that healthcare utilization by the general population increases across all demographics in the United States after age 65 years^4^ and that this is more than would be driven by other age-associated factors such as retirement or social security access.^4^

Elective total knee arthroplasty (TKA) and total hip arthroplasty (THA) are procedures often considered around this Medicare eligibility age.^5^ Importantly, these elective procedures can be initiated or delayed based on patient and surgeon discretion because conservative nonsurgical measures are optimized.^6,7,8,9^

A 2008 article, using data from 1992 to 2003, examined changes in healthcare utilization at 65 years, which included combined elective TKA and THA.^4^ They found that there was a notable increase in the proportion of procedures at 65 years, with a small decrease in utilization at 64 years, suggesting this notion of delaying an elective procedure until the establishment of universal coverage. However, they did not examine the characteristics of those who opted to delay these procedures, only examined combined TKA and THA and used data from before 2003. These data may not be fully applicable at present because the rates of TKA and THA have risen dramatically since then and are expected to continue to rise.^10^

Another 2008 study found an increase in the incidence of elective TKA and THA at age 65 years based on census data.^11^ Here again, there was no focus on the characteristics of those who opted to delay versus those who did not. In addition, this study was conducted in 2008, which compounds the merits for additional follow-up.

This study was conducted to further investigate healthcare utilization for elective TKA and THA around the inflection point of a patient becoming Medicare-eligible and patient factors associated with changes in behavior around this point.

## Methods

### Study Population

The 2012 to 2018 American College of Surgeons National Surgical Quality Improvement Program was used. Patients undergoing elective TKA and THA were identified from this all-payer database based on the Current Procedural Terminology codes 27447 and 27130 with the database indicator of “elective.”

Demographic/comorbidity variables abstracted directly from the data set included age, sex, race/ethnicity, height and weight (used to calculate body mass index [BMI]), American Society of Anesthesiologists (ASA) class, and functional status before surgery. Age was stratified based on Medicare eligibility (<65 years and ≥65 years); BMI was stratified into categories (<18.5, 18.5 to 24, 25 to 29, 30 to 24, and ≥35 kg/m^2^).

Hospital/postoperative variables were tracked for 30 days after the procedures. These included any adverse events (AAEs), serious adverse event (SAE), discharge disposition from hospitalization, and hospital length of stay (LOS).

SAE was identified if there was 30-day incidence of pneumonia, return to the operating room, wound events (infections and dehiscence), sepsis/septic shock, respiratory events (reintubation and failure to wean), thromboembolic events (deep vein thrombosis and pulmonary emboli), cardiac events (myocardial infarction and cardiac arrest), renal events (renal insufficiency and renal failure), stroke/cerebrovascular accident, and death. AAE was identified if there was 30-day incidence of an SAE or any of the following: urinary tract infection, bleeding transfusions, and extended LOS >75th percentile.

### Data Analyses

Initially, the number of combined TKA and THA was plotted by age to assess the age distribution of these procedures. Pearson chi square tests were then used to analyze statistical differences in combined THA and TKA, THA, and TKA when in discrete groups bifurcated at the age of Medicare eligibility (age = 65). Regression discontinuity was conducted using parametric first-order polynomial for all procedures using discrete cutoff of age = 65 and was analyzed using a goodness-of-fit test. This methodology allows us to determine whether there is a notable and unique step-up at Medicare eligibility (age = 65).

The frequency of patient variables and 30-day outcome measures were then tabulated and compared for those younger than 65 years and older than 65 years . Ordinal or categorical variables were compared using Pearson chi square tests. Integer variables (LOS) were treated as continuous and tested for normality using skewness and kurtosis tests and a normal quantile plot. Because LOS was found to be nonparametric, the Mann-Whitney *U* test was used.

Univariate logistic regressions were run for outcome variables *any adverse events* (binary) and *age ≥65 years* (binary, as proxy for a delaying procedure). Categorical predictor variables were compared with a referent.

Multivariate logistic regressions were run for outcome variables using age ≥65 years as proxy for a delaying procedure (Table 5). Categorical predictor variables were compared with a referent. Multivariate models were controlled for all variables determined to be notable in the univariate models. Models were assessed for overall significance and significance of each predictor variable.

Statistical analysis was conducted using Stata v.16.1, and graphs were created using GraphPad Prism 7.0. Statistical significance was defined as *P* ≤ 0.05. This study received an institutional review board exemption because it was determined that there was no research on human subjects.

## Results

### Total Study Population

**Figure 1:**
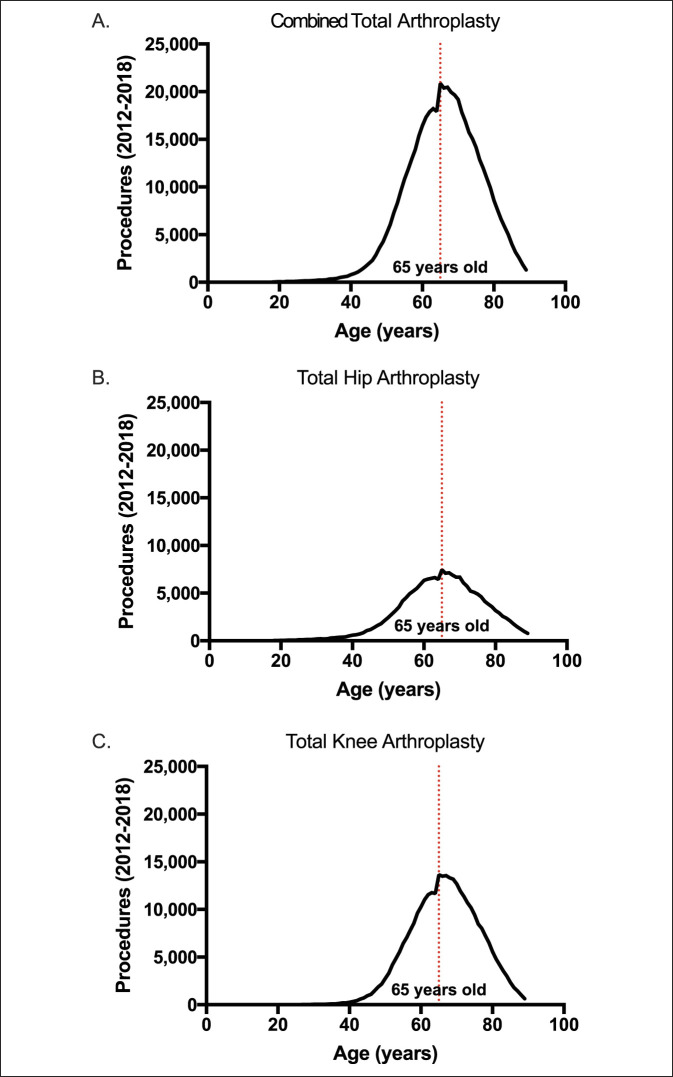
Graphs showing a significant jump at age 65 years in otherwise normally distributed frequency of combined arthroplasty. **A**, Distribution of all individuals undergoing either THA or TKA from 2012 to 2018, plotted as a function of age. Discontinuous jump is noted at age = 65 by the red dashed vertical line. **B**, Distribution of all individuals undergoing THA from 2012 to 2018, plotted as a function of age. Discontinuous jump is noted at age = 65 by the red dashed vertical line. **C**, Distribution of all individuals undergoing TKA from 2012 to 2018, plotted as a function of age. Discontinuous jump is noted at age = 65 by the red dashed vertical line. THA = total hip arthroplasty, TKA = total knee arthroplasty.

In total, 515,139 patients undergoing elective THA or TKA met inclusion criteria for this study, of whom THA = 195,507 (37.96%) and TKA = 319,632 (62.04%). The age distributions were then plotted (Figure 1). Although the overall distribution seemed to follow a classic bell-shaped curve for the combined population, THA, and TKA populations, each seemed to have a discontinuity in the curves at 65 years.

**Figure 2:**
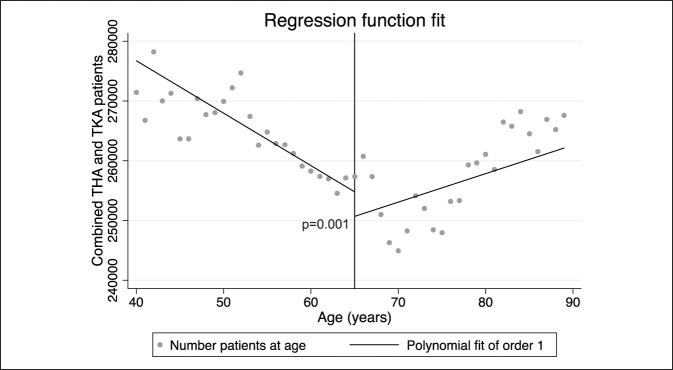
Graph showing significant discontinuous jump in combined total arthroplasty at age 65 years: a first-order polynomial regression discontinuity plot of all individuals undergoing either TKA or THA, with discontinuity cutoff at age = 65 (*P* = 0.001). This demonstrates significant discontinuity at age = 65, indicating that undergoing TKA or THA after 65 years is more likely. THA = total hip arthroplasty, TKA = total knee arthroplasty.

In quantifying the abovenoted discontinuity, the combined THA and TKA population from 63 to 64 years decreased by 1.52%, from 64 to 65 years increased by 15.36%, and from 65 to 66 years decreased by 2.32%. Similar patterns were seen for THA and TKA when evaluated independently. To evaluate this observed discontinuity at age 65 years, the combined THA and TKA groups were plotted on a regression function. Using a first-order polynomial regression with discontinuity at 65 years, there was in fact a significant discontinuity at this age for these elective procedures (*P* = 0.001) (Figure 2).

With the 65-year-old mark established as a discrete divide, the distributions of the population above and below this mark are quantified in Table 1. For the combined population, the mean age was 66.05 ± 10.21 years (mean ± SD).^12^ For THA, this was 64.98 ± 11.32 years, and for TKA, it was 66.70 ± 9.41 years.

**Table 1:** Significantly More Patients Older Than 65 Years Are Having Elective TKA and THA

Groups	<65 yrs, n (%)	≥65 yrs, n (%)	*P* Value
Combined (THA + TKA)			
n = 515,139			
Age = 66.05 ± 10.21 yrs	218,479 (41.41)	296,660 (57.59)	**<0.001**
THA			
n = 195,507			
Age = 64.98 ± 11.32 yrs	90,903 (46.50)	104,604 (53.50)	**<0.001**
TKA			
n = 319,632			
Age = 66.70 ± 9.41 yrs	127,576 (39.91)	192,056 (60.09)	**<0.001**

THA = total hip arthroplasty, TKA = total knee arthroplasty

Significance set at *P* < 0.05.

Age expressed as mean ± SD.

### Focus on the 2 Years Above and Below the Discontinuity at 65 Years

To focus on the discontinuity in utilization for these elective procedures at 65 years, next analyses focused on the 2-year brackets above and below 65 years (63 to 64 years and 65 to 66 years). These groups accounted for a total of 78,182 patients (THA = 27,623 and TKA = 50,559).

The combined THA and TKA 63- to 64-year-old population relative to the 65- to 66-year-old population are characterized in Table 2 to determine whether there were factors associated with who “delayed” surgery. On univariate analyses, those in the 65- to 66-year-old group were more likely to be female (57.64% vs 59.16%; *P* < 0.001), be non-Hispanic White (68.72% vs 71.22%; *P* < 0.001), have BMI ≥35 (34.86% vs 32.59%; *P* < 0.001), and be partially/totally dependent (0.96% vs 1.12%; *P* = 0.035). Notably, those in the 65-66-year-old group were not different in AAE or SAE, but there was a slightly lesser likelihood to go directly home (85.49% vs. 83.12%; p<0.001) and slightly longer LOS (2.42 vs. 2.43 days; p=0.008).

**Table 2:** Combined THA and TKA Show Demographic and Outcome Differences When Stratified by Medicare Eligibility

Variable	63-64	65-66	*P* Value
Total, n (%)	36,511 (46.70)	41,671 (53.30)	**<0.001** ^a^
Sex, n (%)			**<0.001**
Female	21,046 (57.64)	24,653 (59.16)	
Male	15,465 (42.36)	17,018 (40.84)	
Race/ethnicity, n (%)			**<0.001**
Non-Hispanic White	25,090 (68.72)	29,677 (71.22)	
Hispanic	1,196 (3.28)	1,243 (2.98)	
Black or African American	2,696 (7.38)	2,714 (6.51)	
Asian	660 (1.81)	782 (1.88)	
Other	6,869 (18.81)	7,255 (17.41)	
BMI (kg/m^2^), n (%)			**<0.001**
<18.5	132 (0.36)	165 (0.40)	
18.5-24	3,742 (10.25)	4,554 (10.93)	
25-29	9,655 (26.44)	11,331 (27.19)	
30-34	10,255 (28.09)	12,040 (28.89)	
≥35	12,727 (34.86)	13,581 (32.59)	
ASA, n (%)			0.199
1	1,005 (2.75)	1,045 (2.51)	
2	19,512 (53.44)	22,151 (53.16)	
3	15,472 (42.38)	17,871 (42.89)	
4	520 (1.42)	602 (1.45)	
Functional status, n (%)			**0.035**
Independent	36,159 (99.04)	41,025 (98.88)	
Partially/totally dependent	352 (0.96)	466 (1.12)	
AAE, n (%)			0.104
No	30,548 (83.67)	35,044 (84.10)	
Yes	5,963 (16.33)	6,627 (15.90)	
SAE, n (%)			0.302
No	35,329 (96.76)	40,376 (96.89)	
Yes	1,182 (3.24)	1,295 (3.11)	
Discharge disposition, n (%)			**<0.001**
Home	31,215 (85.49)	34,635 (83.12)	
Not home	5,296 (14.51)	7,036 (16.88)	
LOS (d)			**0.008**
Mean ± SD	2.42 ± 1.54	2.43 ± 1.51	
Median (IQR)	2 (2-3)	2 (2-3)	

AAE = any adverse event, ASA = American Society of Anesthesiologists, BMI = body mass index, IQR = interquartile range, LOS = length of stay, SAE = serious adverse event, THA = total hip arthroplasty, TKA = total knee arthroplasty

a Chi square goodness-of-fit test.

Not home includes a skilled care facility, rehab facility, against medical advice, facility that was home, hospice, multilevel senior community, separate acute care, unskilled facility not home, and unknown.

Significance set at *P* < 0.05.

For the THA population, those in the 65- to 66-year-old group were more likely to be female (54.43% vs 56.84%; *P* < 0.001), be non-Hispanic White (71.51% vs 73.24%; *P* < 0.001), have ASA class 3 (37.18% vs 38.40%) or 4 (1.48% vs 1.60%) (*P* = 0.009), and be partially/totally dependent (1.13% vs 1.59%; *P* = 0.001). In addition, those aged 65 to 66 years were less likely to be Black/African American (7.00% vs 6.59%; *P* < 0.001), have BMI ≥35 (23.52% vs 21.55%; *P* = 0.001), have ASA class 1 (3.93% vs 3.30%) or 2 (57.42% vs 56.69%) (*P* = 0.009), or be discharged to home (87.25% vs 84.65%; *P* < 0.001). Notably, those in the 65- to 66-year-old group were more likely to be discharged to a nonhome facility, such as a skilled care facility (7.84% vs 9.41%; *P* < 0.001) or rehab (4.31% vs 5.33%; *P* < 0.001), and have a longer LOS (2.32 vs 2.35; *P* = 0.005) (Table 3).

**Table 3:** THA Shows Demographic, Comorbid, Discharge Destination, and LOS Differences When Stratified by Medicare Eligibility

Variable	63-64	65-66	*P* Value
Total, n (%)	13,080 (47.35)	14,543 (52.65)	**<0.001** ^a^
Sex, n (%)			**<0.001**
Female	7,119 (54.43)	8,252 (56.74)	
Male	5,961 (45.57)	6,291 (43.26)	
Race/ethnicity, n (%)			**0.001**
Non-Hispanic White	9,353 (71.51)	10,651 (73.24)	
Hispanic	276 (2.11)	257 (1.77)	
Black or African American	915 (7.00)	958 (6.59)	
Asian	161 (1.23)	218 (1.50)	
Other	2,375 (18.16)	2,459 (16.91)	
BMI (kg/m^2^), n (%)			**0.001**
<18.5	97 (0.74)	124 (0.85)	
18.5-24	2,225 (17.01)	2,617 (17.99)	
25-29	4,216 (32.23)	4,750 (32.66)	
30-34	3,466 (26.50)	3,918 (26.94)	
≥35	3,076 (23.52)	3,134 (21.55)	
ASA, n (%)			**0.009**
1	514 (3.93)	480 (3.30)	
2	7,510 (57.42)	8,245 (56.69)	
3	4,863 (37.18)	5,585 (38.40)	
4	193 (1.48)	233 (1.60)	
Functional status, n (%)			**0.001**
Independent	12,932 (98.87)	14,312 (98.41)	
Fully/totally dependent	148 (1.13)	231 (1.59)	
AAE, n (%)			0.673
No	10,903 (83.36)	12,150 (83.55)	
Yes	2,177 (16.64)	2,393 (16.45)	
SAE, n (%)			0.521
No	12,653 (96.74)	14,088 (96.87)	
Yes	427 (3.26)	455 (3.13)	
Discharge disposition, n (%)			**<0.001**
Home	11,412 (87.25)	12,310 (84.65)	
Not home	1,668 (12.75)	2,233 (15.35)	
LOS (d)			**0.005**
Mean ± SD	2.32 ± 1.65	2.35 ± 1.62	
Median (IQR)	2 (1-3)	2 (1-3)	

AAE = any adverse event, ASA = American Society of Anesthesiologists, BMI = body mass index, CVA = cerebrovascular accident, DVT = deep vein thrombosis, IQR = interquartile range, LOS = length of stay, OR = operating room, SAE = serious adverse event, THA = total hip arthroplasty, UTI = urinary tract infection

a Chi square goodness-of-fit test.

AAE includes UTI, bleeding transfusions, extended length of stay >75th percentile, pneumonia, return to OR within 30 days, wound events (infections and dehiscence), sepsis/septic shock, respiratory events (reintubation and failure to wean), thromboembolic events (DVT and pulmonary emboli), cardiac events (myocardial infarction and cardiac arrest), renal events (renal insufficiency and renal failure), stroke/CVA, and death.

SAE includes pneumonia, return to OR within 30 days, wound events (infections and dehiscence), sepsis/septic shock, respiratory events (reintubation and failure to wean), thromboembolic events (DVT and pulmonary emboli), cardiac events (myocardial infarction and cardiac arrest), renal events (renal insufficiency and renal failure), stroke/CVA, and death.

Significance set at *P* < 0.05.

For the TKA population, those in the 65- to 66-year-old group were more likely to be female (59.44% vs 60.46%; *P* = 0.02), non-Hispanic White (67.16% vs 70.13%; *P* < 0.001), and discharged to a nonhome facility, such as a skilled care facility (8.88% vs 10.41%; *P* < 0.001) or rehab (5.86% vs 6.50%; *P* < 0.001). In addition, those aged 65 to 66 years were less likely to be Black/African American (7.60% vs 6.47%; *P* < 0.001), have BMI ≥35 (41.19% vs 38.51%; *P* < 0.001), or be discharged to home (84.52% vs 82.30%; *P* < 0.001) (Table 4).

**Table 4:** TKA Shows Demographic and Discharge Destination Differences When Stratified by Medicare Eligibility

Variable	<65 (63-64)	≥65 (65-66)	*P* Value
Total, n (%)	23,431 (46.34)	27,128 (53.66)	**<0.001** ^a^
Sex, n (%)			**0.020**
Female	13,927 (59.44)	16,401 (60.46)	
Male	9,504 (40.56)	10,727 (39.54)	
Race/ethnicity, n (%)			**<0.001**
Non-Hispanic White	15,737 (67.16)	19,026 (70.13)	
Hispanic	920 (3.93)	986 (3.63)	
Black or African American	1,781 (7.60)	1,756 (6.47)	
Asian	499 (2.13)	564 (2.08)	
Other	4,494 (19.18)	4,796 (17.68)	
BMI (kg/m^2^), n (%)			**<0.001**
<18.5	35 (0.15)	41 (0.15)	
18.5-24	1,517 (6.47)	1,937 (7.14)	
25-29	5,439 (23.21)	6,581 (24.26)	
30-34	6,789 (28.97)	8,122 (29.94)	
≥35	9,651 (41.19)	10,447 (38.51)	
ASA, n (%)			0.997
1	491 (2.10)	565 (2.08)	
2	12,002 (51.22)	13,906 (51.26)	
3	10,609 (45.28)	12,286 (45.29)	
4	327 (1.40)	369 (1.36)	
Functional status, n (%)			0.958
Independent	23,227 (99.13)	26,893 (99.13)	
Partially/totally dependent	204 (0.87)	235 (0.87)	
AAE, n (%)			0.091
No	19,645 (83.84)	22,894 (84.39)	
Yes	3,786 (16.16)	4,234 (15.61)	
SAE, n (%)			0.420
No	22,676 (96.78)	26,288 (96.90)	
Yes	755 (3.22)	840 (3.10)	
Discharge disposition, n (%)			**<0.001**
Home	19,803 (84.52)	22,325 (82.30)	
Not home	3,628 (15.48)	4,803 (17.70)	
LOS (d)			0.356
Mean ± SD	2.47 ± 1.47	2.47 ± 1.45	
Median (IQR)	2 (2-3)	2 (2-3)	

AAE = any adverse event, ASA = American Society of Anesthesiologists, BMI = body mass index, CVA = cerebrovascular accident, DVT = deep vein thrombosis, IQR = interquartile range, LOS = length of stay, OR = operating room, SAE = serious adverse event, TKA = total knee arthroplasty, UTI = urinary tract infection

a Chi squared goodness of fit test.

Not home includes a skilled care facility, rehab facility, against medical advice, facility that was home, hospice, multilevel senior community, separate acute care, unskilled facility not home, and unknown.

AAE includes UTI, bleeding transfusions, extended length of stay >75th percentile, pneumonia, return to OR within 30 days, wound events (infections and dehiscence), sepsis/septic shock, respiratory events (reintubation and failure to wean), thromboembolic events (DVT and pulmonary emboli), cardiac events (myocardial infarction and cardiac arrest), renal events (renal insufficiency and renal failure), stroke/CVA, and death.

SAE includes pneumonia, return to OR within 30 days, wound events (infections and dehiscence), sepsis/septic shock, respiratory events (reintubation and failure to wean), thromboembolic events (DVT and pulmonary emboli), cardiac events (myocardial infarction and cardiac arrest), renal events (renal insufficiency and renal failure), stroke/CVA, and death.

Significance set at *P* < 0.05.

### Multivariate Analysis of Predictors for Delaying Elective Total Hip Arthroplasty/Total Knee Arthroplasty

Multivariate analysis was then conducted to assess for predictors of age ≥65 years using THA or TKA (Table 5). This revealed that those who delayed the procedure were more likely to be undergoing TKA (relative to THA, odds ratio [OR], 1.07, *P* < 0.001), be female (OR, 1.08; *P* < 0.001), have BMI ≥35 (OR, 0.87; *P* < 0.001), have ASA >1 (*2* OR, 1.10, *P* = 0.037; *3* OR, 1.17, *P* = 0.001; *4* OR, 1.19, *P* = 0.022), and be partially/totally dependent (OR, 1.17; *P* = 0.026). Relative to non-Hispanic White, other race/ethnicities were less likely to delay (*Hispanic* OR, 0.87, *P* < 0.001; *Black/African American* OR, 0.85; *P* < 0.001; *Others* OR, 0.89; *P* < 0.001).

**Table 5:** Predictors of Age ≥65 Years as Proxy for Delaying Procedures

Variable	Multivariate Model^a^	
	OR (95% CI)	*P* Value
Procedure type		
THA	Referent	**<0.001**
TKA	1.07 (1.04-1.10)	
Female sex	1.08 (1.05-1.11)	**<0.001**
BMI (kg/m^2^)		
<18.5	1.05 (0.83-1.32)	0.675
18.5-24.9	1.03 (0.98-1.09)	0.201
25-29.9	Referent	
30-34.9	0.99 (0.95-1.02)	0.454
≥35	0.87 (0.83-0.90)	**<0.001**
ASA class		
1	Referent	
2	1.10 (1.01-1.20)	**0.037**
3	1.17 (1.07-1.28)	**0.001**
4	1.19 (1.02-1.38)	**0.022**
Functional status		
Independent	Referent	
Partially/totally dependent	1.17 (1.02-1.35)	**0.026**
Race/ethnicity		
Non-Hispanic White	Referent	
Hispanic	0.87 (0.80-0.94)	**0.001**
Black or African American	0.85 (0.80-0.89)	**<0.001**
Asian	0.97 (0.87-1.07)	0.515
Others	0.89 (0.86-0.93)	**<0.001**

ASA = American Society of Anesthesiologists, BMI = body mass index, CI = confidence interval, OR = odds ratio, THA = total hip arthroplasty, TKA = total knee arthroplasty

a Controlled for *all significant variables* from univariate analyses.

Significance set at *P* < 0.05.

## Discussion

At age 65 years, universal health care is established for >98% of the US population through Medicare eligibility^1^ and healthcare utilization has been shown to increase in general at this point.^4^ Although this has been shown specifically for TKA and THA in the past,^4,11^ this study confirms this finding with a contemporary, large, nationally representative database and determines factors associated with this delay.

The bell-shaped distribution curves in this study of TKA and THA (Figure 1) graphically show the discontinuity in age distribution of the utilization of these procedures at 65 years. This spike in utilization at the onset of universal coverage could be evidence of moral hazard. Moral hazard is classically an economic term that explains the phenomenon that individuals alter their behavior in response to someone else bearing the risk of the outcome of that behavior.^13-15^ In medicine, moral hazard is used to state that the availability of health insurance increases spending.^16^ Classically, it states that individuals consume care indiscriminately because they are sheltered from its financial implications. It has been explored through differences in emergency department utilization in teenagers after ineligibility of their parents' health insurance.^17^ It has been shown that the existence of readily available free care shifts individuals' behavior.^15,16^

In general, the characteristics of those who opt to delay the procedure for either TKA or THA are as follows: Individuals who delay are more likely to be female and non-Hispanic White, have a lower functional status, be discharged to a nonhome facility, and have a longer LOS. Interestingly, those older than 65 years undergoing THA were more likely to have an ASA class of >3, but this was not notable in those undergoing TKA or the combined group.

Based on the multivariate analysis for predictors of delaying TKA and THA, factors that were identified included TKA (relative to THA), female sex, BMI ≥35, ASA >1 in a graded fashion, being partially or totally dependent, and race/ethnicity. The implications of these findings may be relevant for those patients in the immediate pre–Medicare-eligibility period contemplating arthroplasty. These merit an additional follow-up because in the future, this could be used to help guide clinical management of these patients.

One could expect that those who delayed their surgery around the time of the 65-year-old transition could be at greater risk of AAEs, but this was not found for SAEs or AAEs after combined or separately assessed TKA or THA. Although discharge to nonhome destination and hospital LOS tended to be statistically significantly longer, the differences were clinically of marginal significance (for combined TKA and THA, discharge to nonhome destination increased by less than 2.5% and LOS increased by an average of 0.01 day).

This study has limitations. This study used data from the National Surgical Quality Improvement Program, and thus, inclusion was based on involvement in this program. The insurance provider was not specifically available, and the 65-year age cutoff was used as a marker of Medicare eligibility. In addition, the factors leading to decisions of when to have surgery were not specifically addressed.

Collectively, these results show that elective arthroplasty is a procedure that a subset of patients close to Medicare eligibility delay until they are 65 years. This provides evidence for moral hazard in medicine because individuals who are likely under universal coverage are more likely to undergo a procedure for which they do not bear financial burden. Differences in the cohorts who delay versus do not delay surgery around this 65-year-old mark also point to healthcare disparities. The demographic, as well as comorbid, differences observed around those who delay versus do not delay should be explored further through the lens of moral hazard and inequitable access across certain demographic brackets.
